# S100A9 extends lifespan in insulin deficiency

**DOI:** 10.1038/s41467-019-11498-x

**Published:** 2019-08-07

**Authors:** Giorgio Ramadori, Sanda Ljubicic, Serena Ricci, Despoina Mikropoulou, Xavier Brenachot, Christelle Veyrat-Durebex, Ebru Aras, Rafael M. Ioris, Jordi Altirriba, Elisabeth Malle, Dirk Foell, Thomas Vogl, Roberto Coppari

**Affiliations:** 1 Diabetes Center of the Faculty of Medicine, University of Geneva, 1211 Geneva 4, Geneva, Switzerland; 2 Department of Cell Physiology and Metabolism, Faculty of Medicine, University of Geneva, 1211 Geneva 4, Geneva, Switzerland; 3 Department of Pediatric Rheumatology and Immunology, University of Munster, 48149 Munster, Germany; 4 Institute of Immunology, University of Munster, 48149 Munster, Germany; 5 Interdisciplinary Centre for Clinical Research, University of Munster, 48149 Munster, Germany; 6 Center for Epigenetics and Metabolism, University of California, Irvine, Irvine, CA 92607 USA

**Keywords:** Type 1 diabetes, Type 1 diabetes

## Abstract

Tens of millions suffer from insulin deficiency (ID); a defect leading to severe metabolic imbalance and death. The only means for management of ID is insulin therapy; yet, this approach is sub-optimal and causes life-threatening hypoglycemia. Hence, ID represents a great medical and societal challenge. Here we report that S100A9, also known as Calgranulin B or Myeloid-Related Protein 14 (MRP14), is a leptin-induced circulating cue exerting beneficial anti-diabetic action. In murine models of ID, enhanced expression of S100A9 alone (i.e. without administered insulin and/or leptin) slightly improves hyperglycemia, and normalizes key metabolic defects (e.g. hyperketonemia, hypertriglyceridemia, and increased hepatic fatty acid oxidation; FAO), and extends lifespan by at least a factor of two. Mechanistically, we report that Toll-Like Receptor 4 (TLR4) is required, at least in part, for the metabolic-improving and pro-survival effects of S100A9. Thus, our data identify the S100A9/TLR4 axis as a putative target for ID care.

## Abstract (web summary)

Insulin replacement is a valuable therapy for insulin deficiency, however, other therapies are being investigated to restore metabolic homeostasis. Here, the authors identify S100A9 as a leptin induced circulating cue that improves glucose and lipid homeostasis and extends survival in insulin deficient mice.

## Introduction

To maintain metabolic homeostasis pancreatic β-cells secrete insulin in response to changes in nutrients level^1–3^. In type 1 diabetes mellitus (T1DM), β-cells are totally (or almost totally) destroyed by an autoimmune-mediated attack^4^. In type 2 diabetes mellitus (T2DM), chronic metabolic pressure leads to β-cells exhaustion, failure, and/or dedifferentiation^5,6^. These defects bring about insulin deficiency (ID)^7^. Life-long insulin therapy is the sole approach currently available to ameliorate patients’ survival and metabolic imbalance^7^. However, maybe due to failure to reach and maintain euglycemia, insulin therapy is unsatisfactory, because insulin deficient patients are at higher risk of developing life-threatening comorbidities (e.g. stroke and heart attack)^8–10^. Of note, more than 100 million people are on suboptimal insulin therapy: these include virtually all T1DM and ~25% of T2DM subjects^11,12^. Thus, it is of paramount medical and societal importance that superior remedies for ID are developed.

Leptin monotherapy (i.e. without the use of administered insulin and/or any other molecule) corrects ID-induced metabolic aberrancies and promotes survival of insulin deficient rodents^13–21^. These results generated great interest in the possibility of treating insulin deficient patients with leptin and/or molecule(s) underlying its beneficial effects. However, development of leptin resistance and the fact that the action of leptin is mainly mediated by hypothalamic neurons^3,16,21–23^ hinders the translational applicability of leptin. Thus, uncovering the downstream mechanisms underlying the beneficial effect of leptin in ID might provide new targets to address this challenge. Hence, with the goal of identifying circulating molecule(s) underlying the advantageous effect of leptin we performed quantitative proteomic analysis of plasma and identified S100A9 as a putative peripheral mediator of leptin action. S100A9 belongs to the EF-hand superfamily of Ca^2+^-binding proteins that forms a complex with S100A8 (S100A8/S100A9 heterodimer; also known as calprotectin) to exert disparate inflammatory effects via Toll-Like Receptor 4 (TLR4)^24,25^ and Receptor of Advanced Glycated End-products (RAGE)^24,26,27^. However, it has been shown that the calgranulins can also exist in monomers to exert anti-inflammatory effects^28^. Noteworthy, S100A9 homodimers have also been reported to directly bind and activate TLR4 and hence promote pro-inflammatory outcomes^29^. Interestingly, S100A9 expression is enhanced in serum and pancreatic islet of T1DM humans; due to the known pro-inflammatory actions of S100A9, this effect is thought to contribute to disease progression^30,31^. Yet, the biological role of S100A9 in metabolism and diabetes is unknown. In this study, we report that enhanced S100A9 in ID mice exerts several beneficial metabolic effects (e.g., it normalizes the hyperketonemia, hypertriglyceridemia, and increased hepatic fatty acid oxidation (FAO) caused by β-cell loss) and extends their lifespan, at least in part, through TLR4 (while other contributing mechanism cannot be ruled out at this stage). Hence, these data unveil a putative approach for improving ID care.

## Results

### S100A9 is as a leptin-induced cue in insulin deficiency

**Fig. 1:**
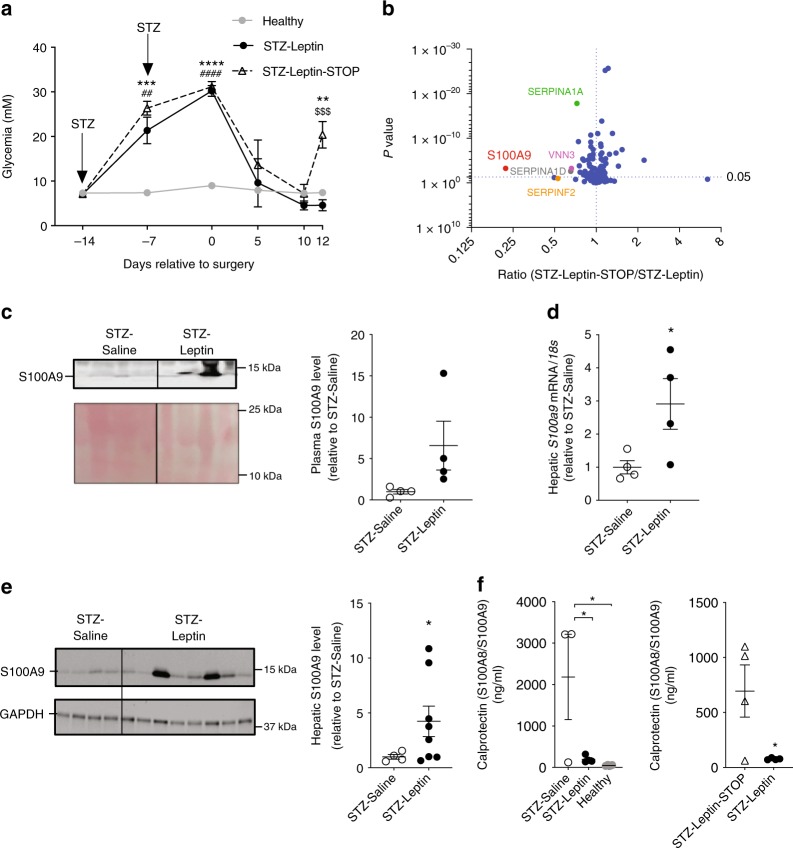
S100A9 is a leptin-induced cue. Intracerebroventricular (icv) delivery of leptin was performed in two groups of STZ-treated, insulin-deficient mice: STZ–Leptin mice received leptin for 12 days; STZ–Leptin–STOP mice received the hormone for 10 days and were followed up to day 12. icv surgery was performed at day 0 and STZ injections were performed 14 and 7 days before surgery. **a** Blood glucose level in STZ–Leptin and STZ–Leptin–STOP mice. The gray line represents average values measured in age-matched non-diabetic healthy control mice. Twelve days after icv surgery, blood samples from STZ–Leptin and STZ–Leptin–STOP were collected and assessed for quantitative proteomic analysis. **b** Volcano plot representing the quantity of each circulating protein detected in STZ–Leptin–STOP divided by the quantity of each respective protein in STZ–Leptin mice. **c** S100A9 content in plasma. **d** *S100a9* mRNA levels. **e** Western blot showing protein content in liver from a cohort of STZ-treated, insulin-deficient mice that underwent either icv leptin or icv saline treatment for 12 days. **f** Calprotectin content in plasma of STZ–Saline, STZ–Leptin, and their healthy controls and in STZ–Leptin and STZ–Leptin–STOP mice. Error bars represent SEM. For all panels *n* = 4/ group, except quantification in **e** that is from four STZ–Saline and eight STZ–Leptin mice. Statistical analyses were done using a two-tailed unpaired Student’s *t-*test when two groups were compared, and one-way ANOVA or two-way ANOVA (Tukey’s post hoc test) when more than two groups and more than one experimental condition/time point were compared, except in **e** where Welch’s *t*-test was used. In **a** ****P < 0.0001, ***P < 0.001, and **P < 0.01 STZ–Leptin–STOP vs Healthy; ^####^*P* < 0.0001 and ^##^*P* < 0.01 STZ–Leptin vs. Healthy; ^$$$^*P* < 0.001 STZ–Leptin–STOP vs Leptin. In **c**–**f** *P < 0.05. Western blot results shown in **c**, **e** are cropped images of the whole gel. Source data are provided as a Source Data file

To identify the mechanisms underlying leptin’s beneficial action, we generated two groups: (i) streptozotocin (STZ)-treated mice that underwent intracerebroventricular (icv) leptin treatment for 12 days (STZ–Leptin) and (ii) STZ-treated mice that underwent icv leptin treatment for 10 days and were withdrawn from leptin treatment for the following two days (STZ–Leptin–STOP). As expected, STZ treatment led to a massive loss of pancreatic insulin-producing β-cells, diminished pancreatic *Proinsulin* mRNA level, and caused severe insulinopenia, and hyperglycemia^13^ (Fig. 1a and Supplementary Fig. 1a–c). As we have previously shown, icv leptin administration did not change the content of pancreatic β-cells, *Proinsulin* mRNA, and plasmatic insulin contents; yet, it greatly reduced hyperglycemia^13,16^ (Fig. 1a and Supplementary Fig. 1a–c). However, two days after leptin delivery was halted hyperglycemia reappeared. Indeed, at this time-point STZ–Leptin–STOP mice had higher circulating glucose content compared to their controls (Fig. 1a). These results demonstrate that STZ–Leptin–STOP and STZ–Leptin mice are insulin deficient and that termination of icv leptin administration leads to reappearance of diabetes symptoms.

We hypothesized that change in plasmatic protein(s) content could underlie re-emergence of hyperglycemia following decrease-of-leptin action (i.e. leptin withdrawal). To identify this change(s), we performed quantitative proteomics analysis. This assay revealed that few circulating proteins including S100A9, Serpina1a, Serpinf2, Serpina1d, and Vanin 3 (VNN3) are downregulated in STZ–Leptin–STOP compared to STZ–Leptin mice (Fig. 1b). Notably, S100A9 was the most reduced plasmatic protein in Leptin–STOP mice (Fig. 1b). These decrease-of-leptin-action results suggest that S100A9 is a leptin-induced cue. To further test this hypothesis, we performed an increase-of-leptin-action experiment by generating STZ-treated mice undergoing icv leptin treatment and their STZ-treated controls receiving icv saline (STZ–Saline). Of note, STZ–Saline and STZ–Leptin mice display a similar degree of severe insulinopenia and leptinopenia^13,16^. In line with our hypothesis, S100A9 plasmatic level was higher in STZ–Leptin compared to STZ–Saline mice (Fig. 1c). Interestingly, while S100A9 expression was unchanged in interscapular brown adipose tissue (iBAT), kidney, and decreased in skeletal muscle, hepatic S100A9 mRNA and protein levels were increased in STZ–Leptin compared to STZ–Saline mice (Fig. 1d, e, and Supplementary Fig. 1d). Our proteomic results also suggested that Serpina1a, Serpinf2, Serpina1d, and VNN3 are leptin-induced cues (Fig. 1b); however, in the abovementioned tissues their expression was not significantly higher in STZ–Leptin compared to STZ–Saline mice (Supplementary Fig. 2a–d). Collectively, these increase- and decrease-of-leptin-action results demonstrate that leptin augments S100A9 content in insulin deficiency.

### S100A9 improves metabolic imbalance in insulin deficiency

**Fig. 2:**
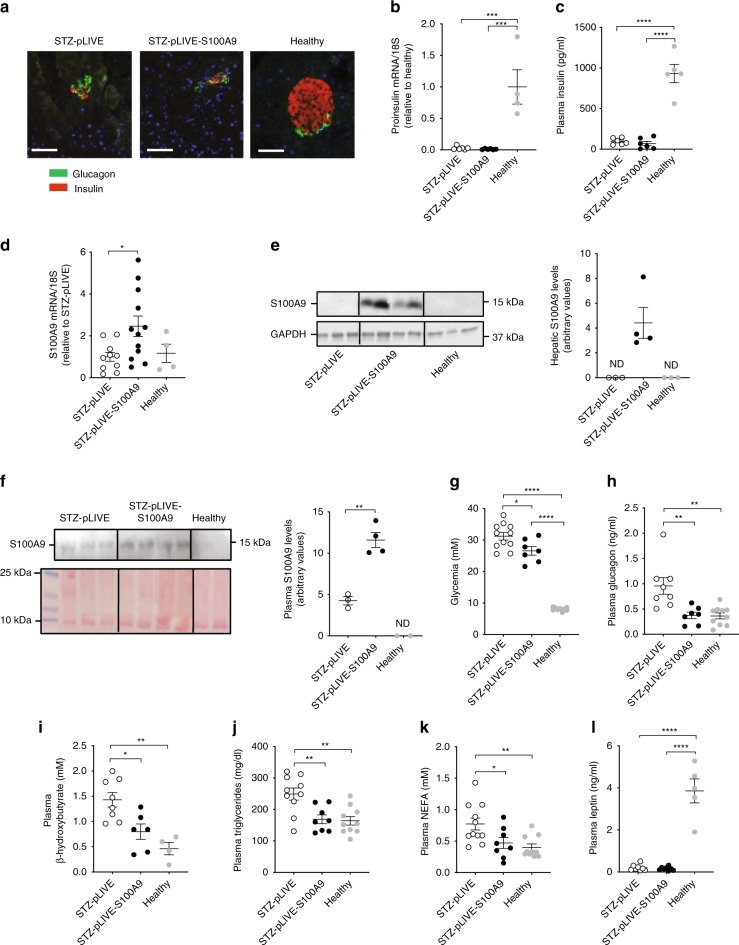
S100A9 ameliorates metabolic imbalance in STZ-induced ID mice. **a** Immunohistochemistry of pancreatic islets showing insulin (red) and glucagon (green) in STZ-treated mice (sacrificed 10 days after HTVI) and their age-matched non-diabetic healthy controls. Scale bar is 50 µm. In STZ-pLIVE and STZ-pLIVE-S100A9 (10 days after HTVI), and their age-matched healthy controls**:** **b** *proinsulin* mRNA (*n*/group = 6, 6, and 4); **c** Plasma insulin content (*n*/group = 6, 6, and 5); **d** hepatic *S100a9* mRNA (*n*/group = 10, 12, and 4); **e** hepatic S100A9 levels (*n* = group: 3, 4, and 3); **f** plasmatic S100A9 levels (*n*/group = 3, 4, and 2); **g** glycemia (*n*/group = 11, 7, and 9); **h** plasma glucagon levels (*n*/group = 8, 7, and 10); **i** plasma β-hydroxybutyrate levels (*n*/group = 8, 6, and 4); **j** plasma triglycerides levels (*n*/groups = 10, 8, and 10); **k** plasma NEFAs levels (*n*/group = 11, 8, and 10); and **l** plasma leptin levels (*n*/group = 8, 8, and 5). Error bars represent SEM. Statistical analyses were done using one-way ANOVA (Tukey’s post hoc test). Of note, similar results were obtained in another cohort maintained under identical conditions. *P < 0.05; **P < 0.01, ***P < 0.001, ****P < 0.0001. Western blot results shown in **e**, **f** are cropped images of the whole g**e**l. Source data are provided as a Source Data file

Our data suggest that leptin-induced S100A9 overexpression exerts beneficial actions; yet, this idea is at odds with the fact that S100A9 forms a complex with S100A8, namely calprotectin, that has been shown to exert several deleterious effects including underlying sepsis-induced lethality^24–28^. Thus, we tested whether ID and/or leptin causes changes in the content of circulating calprotectin. Our data revealed that ID leads to increased plasmatic calprotectin level and that leptin administration normalizes this defect. Indeed, plasmatic calprotectin content was higher and not different in STZ–Saline and STZ–Leptin mice compared to healthy controls, respectively (Fig. 1f). Noteworthy, leptin withdrawal led to reappearance of hypercalprotectinemia as circulating level of calprotectin was elevated in STZ–Leptin–STOP compared to STZ–Leptin mice (Fig. 1f). These increase- and decrease-of-leptin-action results demonstrate that calprotectin is a leptin-suppressed cue. Hence, by diminishing the amount of deleterious calprotectin the increase in S100A9 induced by leptin could lead to beneficial outcomes. To directly test whether S100A9 exerts beneficial actions in ID, we overexpressed S100A9 in two different murine models of ID. As shown in Fig. 1d, e, and Supplementary Fig. 1d, the only tissue in which leptin therapy induced S100A9 expression is the liver (possibly owing to increased S100A9 expression in hepatocytes and/or neutrophils, as these cells are rich in S100A9^32^). Thus, we used hydrodynamic tail vein injection (HTVI; an approach known to target the liver^33^) to deliver either plasmid bearing *S100a9* coding sequences under the control of the albumin promoter (pLIVE-S100A9) or the control empty vector (pLIVE). STZ-treated mice that underwent HTVI of either pLIVE (STZ-pLIVE) or pLIVE-S100A9 (STZ-pLIVE-S100A9) displayed a similar degree of pancreatic β-cells loss, reduced *Proinsulin* mRNA level, and hypoinsulinemia (Fig. 2a–c). To test whether pLIVE-S100A9 HTVI leads to increased S100A9 content we measured hepatic S100A9 mRNA and protein levels and found these parameters to be higher in STZ-pLIVE-S100A9 compared to STZ-pLIVE and healthy mice (Fig. 2d, e). In addition, plasmatic S100A9 level was increased in STZ-pLIVE-S100A9 compared to STZ-pLIVE and healthy mice (Fig. 2f). These results establish that STZ-pLIVE-S100A9 mice are insulin deficient and have increased S100A9 content.

Due to their ID, STZ-pLIVE mice displayed hyperglycemia, hyperglucagonemia, hyperketonemia, hypertriglyceridemia, and increased circulating non-esterified fatty acids (NEFAs) level (Fig. 2g–k). Although STZ-pLIVE-S100A9 mice had hyperglycemia, this parameter was slightly reduced compared to STZ-pLIVE mice (Fig. 2g). Remarkably, glucagonemia, ketonemia, triglyceridemia, and circulating NEFAs contents were all normal in insulin deficient STZ-pLIVE-S100A9 mice (Fig. 2h–k). As predicted, mice lacking insulin were severely hypoleptinemic;^13,16^ yet, this phenotype was not affected by S100A9 overexpression because leptinemia was similarly reduced in STZ-pLIVE-S100A9 and STZ-pLIVE mice compared to healthy controls (Fig. 2l). Altogether, our results suggest that S100A9 overexpression greatly improves metabolic imbalance caused by ID.

**Fig. 3:**
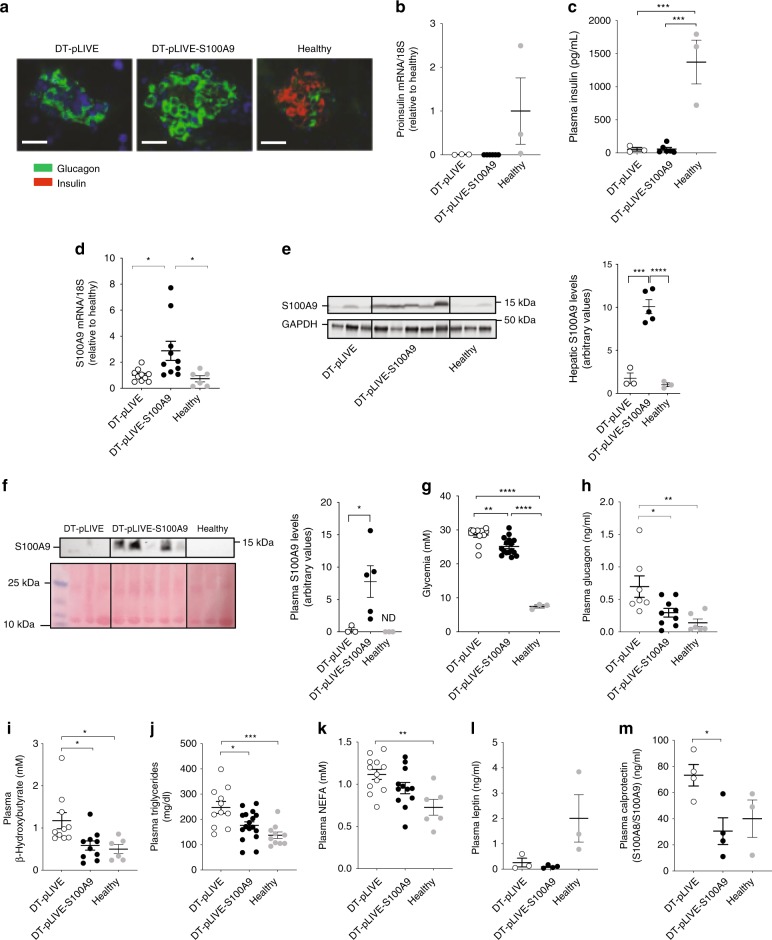
S100A9 ameliorates metabolic imbalance in DT-induced ID mice. **a** Immunohistochemistry of pancreatic islets showing insulin (red) and glucagon (green) in DT-treated *RIP-DTR* mice (sacrificed 10 days after HTVI) and their age-matched non-diabetic healthy controls. Scale-bar is 50 µm. In DT-pLIVE and DT-pLIVE-S100A9 (10 days after HTVI) and their age-matched healthy controls: **b** *proinsulin* mRNA content (*n*/group = 3, 6, and 3); **c** plasma insulin content (*n*/group = 3, 6, and 3); **d** hepatic *S100a9* mRNA (*n*/group = 10, 10, and 6); **e** hepatic S100A9 levels (*n*/group = 3, 5, and 3); **f** plasma S100A9 levels (*n*/group = 3, 5, and 3); **g** glycemia (*n*/group = 11, 15, and 3); **h** plasma glucagon levels (*n*/group = 7, 9, and 6); **i** plasma β-hydroxybutyrate levels (*n*/group = 10, 10, and 6); **j** plasma triglycerides levels (*n*/group = 11, 17, and 10); **k** plasma NEFAs level (*n*/group = 12, 12, and 6); **l** plasma leptin levels (*n*/group = 3, 4, and 3); **m** plasma calprotectin levels (*n*/group = 4, 4, and 3). Error bars represent SEM. Statistical analyses were done using one-way ANOVA (Tukey’s post hoc test). *P < 0.05; **P < 0.01; ***P < 0.001, ****P < 0.0001. Western blot results shown in **f** is a cropped image of the whole gel. Source data are provided as a Source Data file

The STZ model is limited by the fact that it displays minuscule amount of circulating insulin (Fig. 2c); thus, whether this residual insulin exerts biological action in the context of enhanced S100A9 cannot be resolved by studying this model alone. To address this issue we performed HTVI studies on an additional ID model. We used *RIP-DTR* mice that bear a rat insulin promoter (RIP) upstream of diphtheria toxin receptor (DTR) sequences cloned into the *Hprt* locus of the X chromosome. Following three consecutive intraperitoneal DT administrations, *RIP-DTR* mice develop a near-total-loss of pancreatic β-cells^16^. Indeed, almost all of pancreatic β-cells were ablated in DT-injected *RIP-DTR* mice that underwent HTVI of either pLIVE (DT-pLIVE) or pLIVE-S100A9 (DT-pLIVE-S100A9) (Fig. 3a). In line with β-cell loss, pancreatic *Proinsulin* mRNA level was barely measureable and these defects resulted in almost undetectable circulating insulin in DT-pLIVE and DT-pLIVE-S100A9 mice (Fig. 3b, c). To test whether DT-pLIVE-S100A9 mice have increased S100A9 we assessed hepatic S100A9 mRNA and protein levels. These parameters were significantly higher in DT-pLIVE-S100A9 mice compared to DT-pLIVE and healthy controls (Fig. 3d, e). Immunohistochemistry analysis also indicated increased S100A9 in liver of DT-pLIVE-S100A9 compared to DT-pLIVE mice (Supplementary Fig. 3a). In addition, plasmatic S100A9 level was increased in DT-pLIVE-S100A9 mice compared to DT-pLIVE and healthy controls (Fig. 3f). Collectively, these data demonstrate that DT-pLIVE-S100A9 mice are insulin deficient and overexpress S100A9.

Owing to their ID, DT-pLIVE mice developed hyperglycemia, hyperketonemia, hypertriglyceridemia, hyperglucagonemia, hypercalprotectinemia, increased circulating NEFAs level, severe hypoleptinemia, reduced body fat mass, hyperphagia, and polydipsia (Fig. 3g–m and Supplementary Fig 3b–d). Also in line with their ID, DT-pLIVE mice displayed a trend to reduced body weight and lean mass (Supplementary Fig. 3b). Furthermore, we tested whether the pLIVE/HTVI approach influences DT-induced disease. Our data demonstrate that pLIVE/HTVI injection does not affect the level of circulating glucose and β-hydroxybutyrate in DT-treated *RIP-DTR* mice (Supplementary Fig. 3l, m). The outcome of ID on whole-body energy expenditure, substrate utilization, and movement activity is poorly understood^34^; thus, we assessed these parameters. During the dark period, the respiratory exchange ratio (RER) was significantly reduced in DT-pLIVE mice compared to healthy controls (Supplementary Fig. 3e). During the light period, DT-pLIVE mice had increased oxygen consumption resulting in increased energy expenditure compared to healthy controls (Supplementary Fig. 3f–h). Furthermore, in the dark period ambulatory and total movements were diminished in DT-pLIVE compared to healthy mice (Supplementary Fig. 3i, j). These results establish that ID leads to increased energy expenditure and fatty acids and/or amino acids utilization and reduced movements.

Next, we assessed the consequence of S100A9 overexpression on the aforementioned defects. Circulating NEFAs and leptin contents, body weight, fat and lean mass, RER, and ambulatory and total movements were not significantly different between DT-pLIVE-S100A9 and DT-pLIVE mice (Fig. 3k, l; Supplementary Fig. 3b, 3e, 3i, and 3j). Because we observed a trend to reduced food intake in DT-pLIVE-S100A9 compared to DT-pLIVE mice (Supplementary Fig. 3c) we repeated this measurement in another cohort. In this other group, the degree of hyperphagia was not different between DT-pLIVE-S100A9 and DT-pLIVE mice (Supplementary Fig. 3k). Hence, S100A9 overexpression does not affect food intake in ID.

The outcomes of S100A9 overexpression on hyperglycemia and polydipsia were minor as these parameters were only slightly improved in DT-pLIVE-S100A9 compared to DT-pLIVE mice (Fig. 3g and Supplementary Fig. 3d). Remarkably however, the circulating levels of glucagon, calprotectin, β-hydroxybutyrate, and triglycerides and oxygen consumption, and energy expenditure were all similar and significantly reduced between DT-pLIVE-S100A9 mice and healthy and DT-pLIVE controls, respectively (Fig. 3h–j, m and Supplementary Fig. 3f–h).

Similar to leptin therapy (Fig. 1f), S100A9 overexpression normalized the circulating level of calprotectin as this parameter was comparable and significantly reduced between DT-pLIVE-S100A9 mice and healthy and DT-pLIVE controls, respectively (Fig. 3m). To determine the S100A9/S100A8 ratio, we run plasma samples in the same gel and used S100A8 and S100A9 specific antibodies to quantify both proteins. Our data shown in Supplementary Fig. 3n indicate that in samples from ID mice the S100A9/S100A8 ratio is at around 0.68. We were able to detect S100A8 in samples from DT-pLIVE-S100A9 mice in which the S100A9/S100A8 ratio increased to ~0.98 hence indicating excess S100A9 in their plasma. The level of plasmatic S100A8 was also higher in DT-pLIVE-S100A9 vs. DT-pLIVE mice (Supplementary Fig. 3n). Thus, the diminished calprotectin level in DT-pLIVE-S100A9 mice is unlikely due to reduced S100A8. Also, we cannot exclude that the effects of S100A9 overexpression are mediated, at least in part, by increased S100A8.

Based on the profound metabolic effects in ID, we assessed whether S100A9 overexpression influences metabolic homeostasis in the healthy context. Our results shown in Supplementary Fig. 4a–d indicate that in this context the enhanced S100A9 expression does not change food intake, glycemia, and circulating β-hydroxybutyrate and insulin levels. Indeed, mice that underwent HTVI of pLIVE-S100A9 had similar food intake, glycemia, and circulating β-hydroxybutyrate and insulin levels compared to mice that underwent HTVI of pLIVE and healthy mice that did not undergo any treatment (Supplementary Fig. 4a–d). Overall, the aforementioned data gathered from two different models of pancreatic β-cell loss determine that S100A9 overexpression normalizes key metabolic imbalances caused by ID.

### S100A9 suppresses hepatic fatty acid oxidation

A major life-threatening defect in ID is ketoacidosis; in fact, uncontrolled hyperketonemia is thought to underlie the lethality induced by ID^8–10^. As S100A9 overexpression normalizes hyperketonemia in insulin deficient mice, we aimed at unraveling the mechanism underlying this effect. Ketone bodies are synthesized in hepatocytes from acetyl-CoA^35^. In ID, hyperketonemia is majorly sustained by enhanced rate of adipose tissue lipolysis, an event that leads to increased availability of NEFAs as substrate to generate acetyl-CoA from FAO^35,36^. Leptin therapy has been suggested to improve hyperketonemia caused by ID via suppression of the hypothalamic-pituitary-adrenal axis, corticosterone secretion, and adipose tissue lipolysis^20,37^ (a result that has recently been disputed^17^). Thus, S100A9 overexpression could normalize hyperketonemia by suppressing (i) hypercorticosteronemia, (ii) adipose tissue lipolysis, and/or (iii) hepatic FAO.

**Fig. 4:**
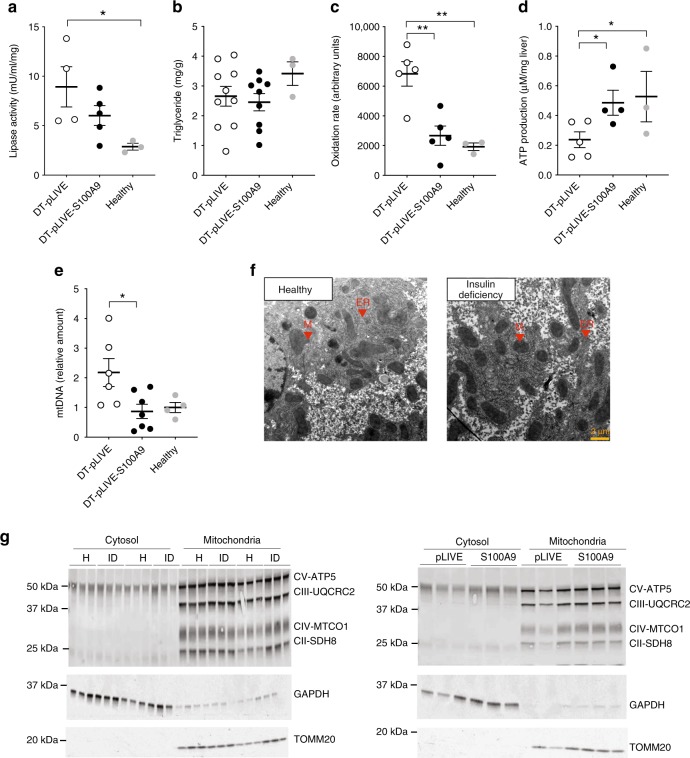
S100A9 dampens hepatic fatty acid oxidation in ID. In DT-pLIVE and DT-pLIVE-S100a9 (10 days after HTVI injection), and their age-matched healthy controls: **a** lipase activity in perigonadal adipose tissue (*n*/group = 4, 5, and 3), **b** hepatic triglycerides (*n*/group = 10, 9, and 3), **c** hepatic fatty acid oxidation rate (using ^14^C-palmitic acid) (*n*/group = 5, 5, and 3), **d** hepatic ATP content (*n*/group = 5, 4, and 3) and **e** hepatic mitochondrial DNA copy number (*n*/group = 5, 6, and 4). **f** Mitochondrial morphology; representative transmission electron microscopy pictures of liver of healthy and insulin deficient mice. M (mitochondria), ER (Endoplasmatic Reticulum). **g** Protein levels of different subunits of oxidative phosphorylation complexes (Complex II = CII, Complex III = CIII, Complex IV = CIV, Complex V = CV) and import receptor TOMM20 in isolated hepatic mitochondria from healthy, insulin deficient (ID), DT-pLIVE and DT-pLIVE-S100A9 mice. Error bars represent SEM. Statistical analyses were done using one-way ANOVA (Tukey’s post hoc test). *P < 0.05; **P < 0.01. Source data are provided as a Source Data file

First, we focused on the putative role of corticosterone and found that icv leptin administration, or withdrawal, does not change the degree of hypercorticosteronemia seen in insulin deficient mice (Supplementary Fig. 5a–b). Also, S100A9 overexpression did not affect hypercorticosteronemia in our two models of ID (Supplementary Fig. 5c–d). Therefore, while reduced corticosterone level might underlie the acute action of leptin^37^ it cannot be a mechanism by which chronic leptin or S100A9 overexpression ameliorates metabolic defects in ID. Second, we assessed adipose tissue lipase activity and found it to be enhanced in insulin deficient DT-pLIVE mice compared to healthy controls (Fig. 4a). Although this parameter tended to be decreased in DT-pLIVE-S100A9 mice, it was not statistically different to DT-pLIVE mice (Fig. 4a). In addition, hepatic triglycerides content was similar between DT-pLIVE-S100A9 mice and their controls (Fig. 4b). Third, we investigated the role of hepatic FAO. In line with their ID^35,36^, DT-pLIVE mice displayed high rate of hepatic FAO compared to healthy controls (Fig. 4c). Remarkably, hepatic FAO rate was normalized in DT-pLIVE-S100A9 mice (Fig. 4c). In keeping with increased FAO and ketogenesis, DT-pLIVE mice had a trend to reduced hepatic ATP level compared to healthy controls (Fig. 4d). Interestingly, S100A9 overexpression normalized this defect; indeed, hepatic ATP content was similar between DT-pLIVE-S100A9 mice and healthy controls (Fig. 4d). Furthermore, while hepatic mitochondrial DNA level was increased in DT-pLIVE mice it was normal in DT-pLIVE-S100A9 mice (Fig. 4e). Hence, due to the importance of mitochondria in ketogenesis, we also measured whether ID leads to changes in hepatic mitochondrial morphology and/or expression of different subunits of oxidative phosphorylation (OXPHOS) complexes and mitochondrial import receptor TOMM20. Surprisingly, mitochondrial morphology was normal in insulin deficient mice (Fig. 4f). In addition, hepatic contents of TOMM20 and diverse OXPHOS subunits were unaffected by ID and S100A9 overexpression (Fig. 4g*)*. Collectively, our results demonstrate that S100A9 overexpression normalizes the increased hepatic FAO caused by ID.

### S100A9 reduces hyperketonemia and prolongs lifespan via TLR4

**Fig. 5:**
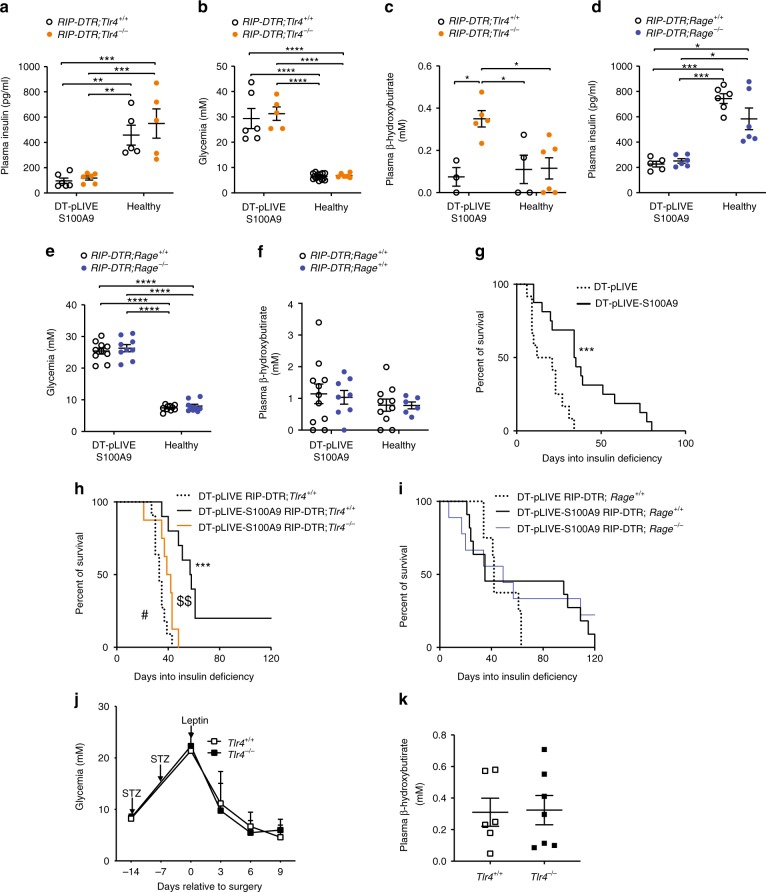
TLR4 is required for the action of S100A9 on hyperketonemia and survival in ID. In DT-treated pLIVE-S100A9 *RIP-DTR*; *Tlr4*^*+/+*^-, DT-treated pLIVE-S100A9 *RIP-DTR*; *Tlr4*^−*/−*^-, DT-untreated (healthy) *RIP-DTR*; *Tlr4*^*+/+*^-, and DT-untreated (healthy) pLIVE-S100A9 *RIP-DTR*; *Tlr4*^−*/*−^ mice: **a** plasma insulin level (*n*/group = 6, 6, 5, and 5); **b** glycemia (*n*/group = 6, 5, 15, and 6), and **c** plasma β-hydroxybutyrate content (*n*/group = 3, 5, 4, and 6). In DT-treated pLIVE-S100A9 *RIP-DTR*; *Rage*^*+/+*^-, DT-treated pLIVE-S100A9 *RIP-DTR*; *Rage*^*−/−*^-, DT-untreated (healthy) *RIP-DTR*; *Rage*^*+/+*^-, and DT-untreated (healthy) pLIVE-S100A9 *RIP-DTR*; *Rage*^*−/−*^ mice: **d** plasma insulin level,(*n*/group = 5, 6, 6, and 6); **e** glycemia (*n*/group = 10, 9, 11, and 9) and **f** plasma β-hydroxybutyrate content (*n*/group = 11, 8, 10, and 6). Error bars represent SEM. Statistical analyses have been performed by two-way ANOVA (Tukey’s post hoc test) *P < 0.05; **P < 0.01; ***P < 0.001; ****P < 0.0001. Kaplan–Meier survival analyses (**g**–**i**). According to Log-Rank Mantel Cox test as well as Gehan-Beslow Wilcoxon test, DT-pLIVE-S100A9 mice (*n* = 16) have higher survival as compared to pLIVE controls (*n* = 12) (**g**). DT-pLIVE-S100A9 *Tlr4*^*−*^^*/−*^ (*n* = 9) have similar lifespan as DT-pLIVE *Tlr4*^*+/+*^ controls (*n* = 11) while DT-pLIVE-S100A9 *Tlr4*^*+/+*^ (*n* = 11) have higher survival compared to the latter (**h**). DT-pLIVE-S100A9 *Rage*^*−/−*^ (*n* = 9) and DT-pLIVE-S100A9 *Rage*^*+/+*^ (*n* = 11) have similar lifespan which is extended compared to DT-pLIVE *Rage*^*+/+*^ controls (*n* = 8) (**i**). In **g** ***P < 0.001 DT-pLIVE mice vs DT-pLIVE-S100A9 mice. In **h** ***P < 0.001; DT-pLIVE-S100A9 *RIP-DTR*; *Tlr4*^*+/+*^ mice vs. DT-pLIVE *RIP-DTR*; *Tlr4*^*+/+*^ mice; ^$$^*P* < 0.01; DT-pLIVE-S100A9 *RIP-DTR*; *Tlr*^−*/−*^ mice vs. DT-pLIVE-S100A9 *RIP-DTR*; *Tlr4*^*+/+*^ mice; ^#^*P* < 0.05; DT-pLIVE-S100A9 *RIP-DTR*; *Tlr4*^*−/−*^ mice vs. DT-treated pLIVE *RIP-DTR*; *Tlr4*^*+/+*^ mice. Circulating glucose **j** and β-hydroxybutyrate **k** content in DT-treated *RIP-DTR*; *Tlr4*^*−/*−^ (*n* = 7) and *RIP-DTR*; *Tlr4*^*+/+*^ (*n* = 6) mice after icv leptin therapy. Statistical analysis in **k** was done using a two-tailed unpaired Student’s *t-*test. Source data are provided as a Source Data file

To determine the molecular mechanism underlying the metabolic actions of S100A9 we directly tested the relevance of its putative receptors: TLR4 and RAGE^24,26–28^. To this end, we crossed the *Rage* null^24^ with the *RIP-DTR* allele and generated *RIP-DTR* males that were homozygous for either the *Rage* null (*RIP-DTR*; *Rage*^−*/−*^ mice) or the *Rage* wild-type (*RIP-DTR*; *Rage*^*+/+*^ mice) allele. Also, we crossed the *Tlr4* null^38^ with the *RIP-DTR* allele and generated *RIP-DTR* males that were homozygous for either the *Tlr4* null (*RIP-DTR*; *Tlr4*^*−/−*^ mice) or the *Tlr4* wild-type (*RIP-DTR*; *Tlr4*^*+/+*^ mice) allele. DT administration caused a similar degree of severe hypoinsulinemia in *RIP-DTR*; *Tlr4*^*−/−*^ and *RIP-DTR*; *Rage*^−*/*−^ mice and their littermate controls with intact TLR4 and RAGE, respectively, and S100A9 overexpression did not affect this parameter (Fig. 5a, d). Likewise, DT administration led to a similar level of hyperglycemia in *RIP-DTR*; *Tlr4*^*−/−*^ and *RIP-DTR*; *Rage*^*−/−*^ mice and their littermate controls with intact TLR4 and RAGE, respectively, and S100A9 overexpression did not affect this parameter (Fig. 5b, e). On the other hand, whereas insulin deficient mice overexpressing S100A9 and lacking RAGE and their intact controls had normal circulating β-hydroxybutyrate level (Fig. 5f), S100A9 overexpression failed to reduce this parameter in insulin deficient mice lacking TLR4 (Fig. 5c). Thus, while RAGE is dispensable TLR4 is required for the effects of S100A9 in normalizing hyperketonemia.

ID rapidly leads to lethality. Thus, we tested whether S100A9 overexpression improves lifespan in ID. As expected, DT-pLIVE mice were short-lived as 50% of them were able to survive only up to 16.5 days and all others inevitably succumbed within 34 days after DT administration (Fig. 5g). On the other hand, median and maximal lifespan of DT-pLIVE-S100A9 was 34.5 and 80 days into ID (Fig. 5g). Next, we directly tested whether RAGE and/or TLR4 is required for the pro-survival effect of S100A9. Also in these different genetic backgrounds, S100A9 overexpression greatly extended lifespan in ID (DT-pLIVE-S100A9 *RIP-DTR*; *Tlr4*^*+/+*^ vs. DT-pLIVE *RIP-DTR*; *Tlr4*^*+/+*^ mice and DT-pLIVE-S100A9 *RIP-DTR*; *Rage*^*+/+*^ vs. DT-pLIVE *RIP-DTR*; *Rage*^*+/+*^mice; Fig. 5h, i). However, while S100A9 overexpression exerted similar pro-survival action in insulin deficient mice lacking RAGE (DT-pLIVE-S100A9 *RIP-DTR*; *Rage*^*+/+*^ vs. DT-pLIVE-S100A9 *RIP-DTR*; *Rage*^*−/−*^ mice; Fig. 5i) it failed to promote survival in insulin deficient mice lacking TLR4 (DT-pLIVE-S100A9 *RIP-DTR*; *Tlr4*^*+/+*^ vs. DT-pLIVE-S100A9 *RIP-DTR*; *Tlr4*^*−/−*^ mice; Fig. 5h). Thus, TLR4 is required for the metabolic-improving and pro-survival actions of S100A9.

As S100A9 is a leptin-induced cue, these results suggest that TLR4 is also required for the beneficial action of leptin. To directly test this hypothesis, we assessed the outcome of leptin administration in insulin deficient mice lacking TLR4. As expected, leptin therapy improved the hyperglycemia and hyperketonemia caused by ID in *Tlr4*^*+/+*^ controls; yet, similar outcomes were observed in insulin deficient *Tlr4*^*−/−*^ mice (Fig. 5j, k). Collectively, our data demonstrate that although TLR4 is dispensable for the action of leptin, this receptor is required for the beneficial effect of S100A9 in ID.

## Discussion

Here we show that S100A9 is a leptin-induced cue exerting several protective actions against metabolic imbalance and lethality caused by ID. For example, while its effect on hyperglycemia is minor our data indicate that S100A9 overexpression is able to normalize enhanced hepatic FAO and hyperketonemia and significantly promote survival in different cohorts of insulin deficient mice. Of note, all of these beneficial effects were achieved in the context of ID and severe hypoleptinemia and without administration of either insulin or leptin. As such, our data unmask a molecular mechanism that beyond leptin and insulin promotes beneficial metabolic and pro-survival actions in ID.

It is important to mention that the ID models used in this study have their own inherent limitations. For example, in addition to pancreatic β-cells the *RIP* promoter is also active in hypothalamus of *RIP-DTR* mice^16^. Therefore, the possibility that DT administration destroys hypothalamic cells in *RIP-DTR* mice is plausible. On the other hand, the STZ model is imperfect due to incomplete pancreatic β-cell ablation^13^. To overcome these caveats, we tested the effects of S100A9 overexpression in both models. As our data indicate that S100A9 overexpression exerts similar beneficial effects in the DT- and STZ-induced models, we suggest that our results are not secondary or driven by a specific ID model but rather caused by S100A9 action. In addition, because changes in food intake could influence the defects caused by ID it was important to determine whether the beneficial action of S100A9 is secondary to caloric intake alterations. Thus, we measured food intake in several cohorts and found that mice overexpressing S100A9 do not display altered food intake. However, after a few days of ID the vast majority of DT-pLIVE controls is already dead and only very few of them are able to survive up for a month. Thus, comparative analysis of cumulative food intake over an extended period of time between DT-pLIVE-S100A9 mice and their controls is not possible. Consequently, we cannot rule out the possibility that over time small differences in food intake might contribute to the metabolic-improving and pro-survival actions of S100A9.

The encouraging pre-clinical results showing efficacy of leptin monotherapy in ID^13–21^ spurred great clinical interest. However, a recent study by Garg and colleagues indicated little effect of leptin in eight T1DM humans^39^. Of note, differently from murine models of ID showing severe hypoleptinemia^13,16^ these subjects displayed normal circulating leptin levels^39^. Thus, even though this was a small clinical trial these results are in line with previous human studies indicating that regardless to the disease context (e.g. lipodystrophy, hypothalamic amenorrhea) leptin therapy is efficacious only in severely hypoleptinemic subjects^40–44^. Therefore, to determine the clinical significance of leptin therapy in ID larger clinical trials enrolling T1DM subjects with low circulating leptin levels (e.g. young and lean individuals) are warranted. Nevertheless, it is formally possible that development of leptin resistance renders leptin therapy unsatisfactory. Because we identified S100A9/TLR4 as a downstream axis of leptin action, we suggest that this mechanism might represent a molecular target for approaches able to circumvent the plausible leptin resistant restraint. Yet, the role of S100A9 in human insulin deficiency has not been tested and therefore is unknown.

Our findings are of further human relevance as S100A9 expression is increased in patients with ID^30,31,45^. Because S100A9 exerts pro-inflammatory actions^24–29,45^ its increase in T1DM patients might be seen as a contribution to disease development. Contrariwise, our data reveal a completely unanticipated role of S100A9 as they suggest that augmented S100A9 is a pathophysiological adaptation against disease progression; an effect that could be exploited therapeutically. In addition to diabetes, our findings are relevant in the context of other serious maladies. Indeed, increased calprotectin has been described in life-threatening conditions where it promotes for example development of systemic autoimmunity and sepsis-induced lethality^24,25^. Yet, means to dampen calprotectin are lacking. Here we show that enhanced S100A9, or leptin, diminishes calprotectin. Thus, we propose that S100A9 and leptin represent tools to reduce calprotectin with putative beneficial outcomes in several maladies, including sepsis and autoimmunity. To test this idea, future studies are necessary.

Mechanistically, by testing the effects of S100A9 overexpression in mice devoid of RAGE or TLR4 we established the relevance of these receptors. Our data demonstrate that while the former is dispensable the latter is key for the pro-survival and metabolic-improving action of S100A9. However, we must acknowledge that results gathered from genetically-engineered mice lacking a given gene could be confounded by the inherent limitations of these animal models (e.g. compensatory mechanisms). Also, whether TLR4 expressed by hepatocytes (and/or other cells) mediates the effects of S100A9 is still unknown. Future studies aimed at testing the effects of S100A9 in ID mice expressing Cre-recombinase in a cell-type-specific manner (e.g. hepatocytes, or pancreatic alpha cells) and bearing Cre-conditional *Tlr4* alleles will be required. Results from these experiments will determine whether TLR4 in hepatocytes (and/or other cells) is required and/or sufficient for mediating the effects of S100A9 in ID. In addition, based on these loss-of-function results we performed additional mechanistic experiments. To directly test the role of TLR4 in mediating the action of leptin we delivered icv leptin in insulin deficient mice lacking TLR4 and their controls. Interestingly, our data identify an unexpected redundancy in leptin action. In fact, while leptin-induced S100A9/TLR4 signaling appears to be sufficient this pathway is not required for the beneficial metabolic action of leptin. Therefore, in ID leptin therapy improves metabolic defects (e.g. hyperketonemia) by TLR4-dependent (e.g. by increasing S100A9) and -independent (yet-to-be-identified) mechanisms. In summary, our data uncover an unexpected pathway that, beyond leptin and insulin, improves metabolism and promotes survival in ID and identify S100A9/TLR4 axis as a putative target for treatment of maladies caused by β-cell loss.

## Methods

### Animals and induction of insulin-deficiency

All mice were maintained with standard chow diet and water available ad libitum in a light- and temperature-controlled environment. All the experiments described in the study used adult male mice. Two insulin deficient animal models were generated and studied. Model 1: Diphtheria Toxin (DT, Sigma Aldrich) was dissolved in sterile 0.9% NaCl and intraperitoneally administrated into *RIP-DTR* animals (provided by Dr. P. Herrera)^16^ (0.5 μg/kg body weight at days 0, 1, 4). Model 2: Streptozotocin (STZ, Sigma Aldrich) was dissolved in sterile 0.9% NaCl and intraperitoneally administrated (150 mg/kg body weight two times at one week intervals) as previously described^13^. *RIP-DTR*; *Tlr4*^*−/−*^ male mice and their littermates *RIP-DTR*; *Tlr4*^*+/+*^ male controls were generated by breeding males that were heterozygous for the *RIP-DTR* allele and heterozygous for the *Tlr4* null allele^38^ (*Tlr4* null mice were provided by Drs. S. Fabbiano and M. Trajkovski) with females that were homozygous for the *RIP-DTR* allele and heterozygous for the *Tlr4* null allele. From these breeding pairs we obtained 100% males bearing the *RIP-DTR* allele of which 25% were homozygous for the *Tlr4* null allele (*RIP-DTR*; *Tlr4*^*−/−*^ mice) and 25% were homozygous for the *Tlr4* wild-type allele (*RIP-DTR*; *Tlr4*^*+/+*^ mice). *RIP-DTR*; *Rage*^*−/−*^ male mice and their littermates *RIP-DTR*; *Rage*^*+/+*^ male controls were generated by breeding males that were heterozygous for the *RIP-DTR* allele and heterozygous for the *Rage* null allele (*Rage* null mice were provided by Dr. D. Foell)^24^ with females that were homozygous for the *RIP-DTR* allele and heterozygous for the *Rage* null allele. From these breeding pairs we obtained 100% males bearing the *RIP-DTR* allele of which 25% were homozygous for the *Rage* null allele (*RIP-DTR*; *Rage*^*−/−*^ mice) and 25% were homozygous for the *Rage* wild-type allele (*RIP-DTR*; *Rage*^*+/+*^ mice). Care of mice at University of Geneva was within the procedures approved by animal care and experimentation authorities of the Canton of Geneva, Switzerland (animal protocol numbers GE/22/15, GE/28/13, GE/120/15, GE/78/18).

### Leptin intracerebroventricular treatment

A cannula was positioned stereotaxically into the cerebral lateral ventricles (−0.34 mm from the bregma, ±1 mm lateral, −2.5 mm from the skull), and a small osmotic minipump (model 1004; Alzet) implanted s.c. was attached via a catheter to the cannula for icv infusion. The catheter was 25% longer than the distance between the site of placement of the minipump and the cannula to allow free movement of the neck. The minipump was filled with placebo or leptin at a final concentration of 227 ng/μL. Because a volume of 0.11 μL/h was infused, these mice received 25 ng of icv leptin per hour as previously described^13^. For STZ–Leptin–STOP animals, leptin therapy was halted at day 10 after icv surgery by cutting and sealing the catheter linking the osmotic minipump to the brain cannula.

### Assessment of energy homeostasis

Energy expenditure and the respiratory exchange ratio were determined by indirect calorimetry, locomotor activity was recorded by an infrared frame, and food and fluid intake were measured by highly sensitive feeding and drinking sensors. These parameters were measured in mice housed individually in Labmaster metabolic cages (TSE, Bad Homburg, Germany) at the Small Animal Phenotyping Core Facility (University of Geneva, Geneva, Switzerland) under controlled temperature (22 ± 1 °C) and light/dark cycles (light on: 7 AM–7 PM). An EchoMRI-700 quantitative nuclear magnetic resonance analyzer (Echo Medical Systems, Houston, USA) was used to measure body composition.

### Plasma proteomics analysis

At the Proteomics Core Facility (University of Geneva, Geneva, Switzerland) plasma samples were individually digested with trypsin and the resulting peptides mixtures were then labeled with one of the eight iTRAQ label reagents. Samples were then pooled and fractionated in 12 fractions by off-gel electrophoresis (i.e. peptides were separated according to their isoelectric point). Each fraction was then analyzed by reverse-phase LC-MS/MS on a quadrupole linear trap (LTQ) orbitrap Velos Pro mass spectrometer coupled in-line with a nanoAcquity UPLC. MS raw data from all the fractions were converted to mgf peaklist, merged, and searched against the uniprot mouse database using EasyProt software platform. Quantitative proteomics were obtained using the embedded IsoQuant module. The mass spectrometry proteomics data have been deposited to the ProteomeXchange Consortium via the PRIDE partner repository with the dataset identifier PXD013466.

### Assessment of mRNA, protein, and substrates content

Mice were sacrificed and their tissues quickly removed and snap-frozen in liquid nitrogen and subsequently stored at −80 °C. RNAs were extracted using Trizol reagent (Invitrogen). Complementary DNA was generated by Superscript II (Invitrogen) and used with SYBR Green PCR master mix (Applied Biosystem, Foster City, CA, USA) for quantitative real time PCR (q-RTPCR) analysis. mRNA contents were normalized to 18s mRNA levels (Supplementary Table 1 shows the list of primers used herein). All assays were performed using an Applied Biosystems QuantStudio® 5 Real-Time PCR System. For each mRNA assessment, q-RTPCR analyses were repeated at least three times. Proteins were extracted by homogenizing samples in lysis buffer (Tris 20 mM, EDTA 5 mM, NP40 1% (v/v), protease inhibitors (P2714-1BTL from Sigma, St. Louis, MO, USA), then resolved by SDS-PAGE and finally transferred to a nitrocellulose membrane by electroblotting. The following antibodies were used: CalgranulinB-S100A9 (cat. Number PB9678, Boster; dilution 1:500) and GAPDH (14c10, #2118, Cell Signalling; dilution 1:2000). An in-house ELISA (designed as follows: an antibody specific for S100A8 was used for capturing and an antibody specific for S100A9 was used for detection) as previously described^46^ determined murine S100A8/S100A9 (calprotectin) amounts. Hepatic triglycerides levels were measured as previously described^13^. Unprocessed western blot scans are shown in Source Data File.

### Circulating substrates and hormones levels

Tail vein blood was collected between 2 and 4 PM from mice that were fed ad libitum. To avoid random post-prandial confounding effects food was removed 2 h prior to blood collection. Serum or plasma samples were collected after centrifugation (3500 × *g*, 10 min) and stored at −80 °C. Glucose, non-esterified fatty acids, triglycerides, ketone bodies, glucagon, corticosterone, and leptin levels were measured using commercially available kits as already described^13,47^.

### Immunofluorescence and immunohistochemistry

For determining pancreatic islets anatomy and glucagon and insulin distribution pancreata were fixed using Formalin 4%, embedded in Parrafin and cut in 15-μm-thick sections using a cryostat. Sections were incubated with primary antibodies for 12 h at room temperature. Primary antibodies used were: cobaye anti-swine insulin (Dako A0564; dilution 1:400) and rabbit anti-glucagon (Abcam ab92517; dilution 1:100). Secondary antibodies used were Alexa Fluor 488 donkey anti-rabbit IgG (Molecular Probes A11008; dilution 1:400) and Alexa Fluor 594 goat anti-cobaye IgG (Molecular Probes A11076; dilution 1:400). S100A9 immunohistochemistry was performed in liver slides cut at 5 µm thickness. Slides were incubated in the Rabbit S100A9 antibody (diluted at 1/500; cat. Number PB9678, Boster)) overnight and then incubated with a biotinyated antibody anti-Rabbit (diluted at 1/100) and revealed by adding Vectsatin and DAB. Slides were mounted and colored with hematoxylin (to reveal nuclei).

### Overexpression of S100A9

Hydrodynamic tail vein injection (HTVI) was performed according to a previously described method^48^. Overexpression of S100A9 was achieved by using pLIVE vectors (Myrus) that allow expression of a given gene under the control of the albumin promoter. The following sequences were cloned into pLIVE between restriction sites BamH1 and Xho1:

GCTAGCGGATCCGCCGCCACCATGGCCAACAAAGCACCTTCTCAGATGGAGCGCAGCATAACCACCATCATCGACACCTTCCATCAATACTCTAGGAAGGAAGGACACCCTGACACCCTGAGCAAGAAGGAATTCAGACAAATGGTGGAAGCACAGTTGGCAACCTTTATGAAGAAAGAGAAGAGAAATGAAGCCCTCATAAATGACATCATGGAGGACCTGGACACAAACCAGGACAATCAGCTGAGCTTTGAGGAGTGTATGATGCTGATGGCAAAGTTGATCTTTGCCTGTCATGAGAAGCTGCATGAGAACAACCCACGTGGGCATGGCCACAGTCATGGCAAAGGCTGTGGGAAGGACTACAAAGACGATGACGACAAGTGACTCGAG.

pLIVE-S100A9 plasmid DNA was sequenced to confirm correct sequences and orientation. Each mouse received 50 μg of pLIVE-S100A9 or pLIVE; aged-matched mice that did not undergo any procedure were used as healthy controls.

### Assessment of ex vivo FAO rate and ATP level

The ex vivo radioactive fatty acid oxidation measurements were performed by using ^14^C-palmitic acid following an established protocol^49^. Before radioactive measurements, the lysis was done starting from 250 mg of liver in 0.5 ml of lysis buffer, and 50 μl of lysate in addition to 150 μl total FAO reaction buffer. The reaction is performed at 37 °C × 60′. For ATP levels assessment, a piece of liver was used and the metabolite was measured by using the ATP fluorimetric assay kit from Sigma.

### Adipose tissue lipase activity

Lipase activity rate was measured in the perigonadal white adipose tissue (pWAT) by using the lipase activity colorimetric kit assay II (Biovision). Briefly, 50 mg of pWAT was homogenized in 4 volumes of Assay Buffer and centrifuged (13,000 × *g*, 10 min) to remove insoluble material. Samples were then diluted in the Assay Buffer and lipase activity was measured at 412 nm, using a standard curve, a positive and a negative control provided by the kit assay.

### Mitochondrial study

mtDNA analysis was performed as described before^50^. Briefly, total DNA was extracted with DNeasy blood and tissue kit (QIAGEN). mtDNA was amplified using primers specific for the mitochondrial cytochrome c oxidase subunit 2 (COX2) gene (Forward ATAACCGAGTCGTTCTGCCAAT; Reverse TTTCAGAGCATTGGCCATAGAA) and normalized to genomic DNA by amplification of the ribosomal protein s18 (rps18) nuclear gene (Forward TGTGTTAGGGGACTGGTGGACA; Reverse CATCACCCACTTACCCCCAAAA). q-RTPCR reactions were performed using SYBR Green PCR master mix (Applied Biosystem, Foster City, CA, USA) and Applied Biosystems QuantStudio® 5 Real-Time PCR System. Mitochondria isolation from liver tissues was performed as described before^51,52^. Isolated mitochondria were analyzed in SDS-PAGE. Detection of different oxidative phosphorylation subunits and import proteins was achieved by using a total OXPHOS rodent WB antibody cocktail (ab110413, Abcam; dilution 1:1000) and TOMM20 (ab56783, Abcam; dilution 1:1000), respectively. Liver tissue samples were fixed in 0.15 M Cacodylate, 2 mM CaCl_2_, 2%, Perfluoroalkoxy alkane and 2.5% glutaraldehyde. Speciments were prepared with a standard dehydratation and epon-embedding and sectioning of tissues method. Speciments were analyzed by using Morgagni Transmission electron microscopy and iTEM software.

### Statistical analysis

Data sets were analyzed for statistical significance using PRISM (GraphPad, San Diego, CA) for a two-tail unpaired Student’s *t*-test when two groups were compared or one-or two-way ANOVA (Tukey’s post test) when more than two groups were compared.

### Reporting summary

Further information on research design is available in the Nature Research Reporting Summary linked to this article.

## Supplementary information

Supplementary Information

Reporting Summary

Source Data

## Data Availability

Data generated or analyses during this study are either included in this published article (and its supplementary information files and data source file) or available from the corresponding author on reasonable request. The mass spectrometry proteomics data that support the findings of this study have been deposited to the ProteomeXchange Consortium via the PRIDE^52^ partner repository with the dataset identifier PXD013466.
