# Supplementary material for: S100A9 extends lifespan in insulin deficiency
Source: Nat Commun. 2019 Aug 7;10:3545. doi: 10.1038/s41467-019-11498-x (PMC6686003; doi:10.1038/s41467-019-11498-x)
Supplement: Supplementary file 1 — Supplementary Information [file 41467_2019_11498_MOESM1_ESM.pdf]

S100A9 extends lifespan in insulin deficiency

Supplementary information

Ramadori et al.

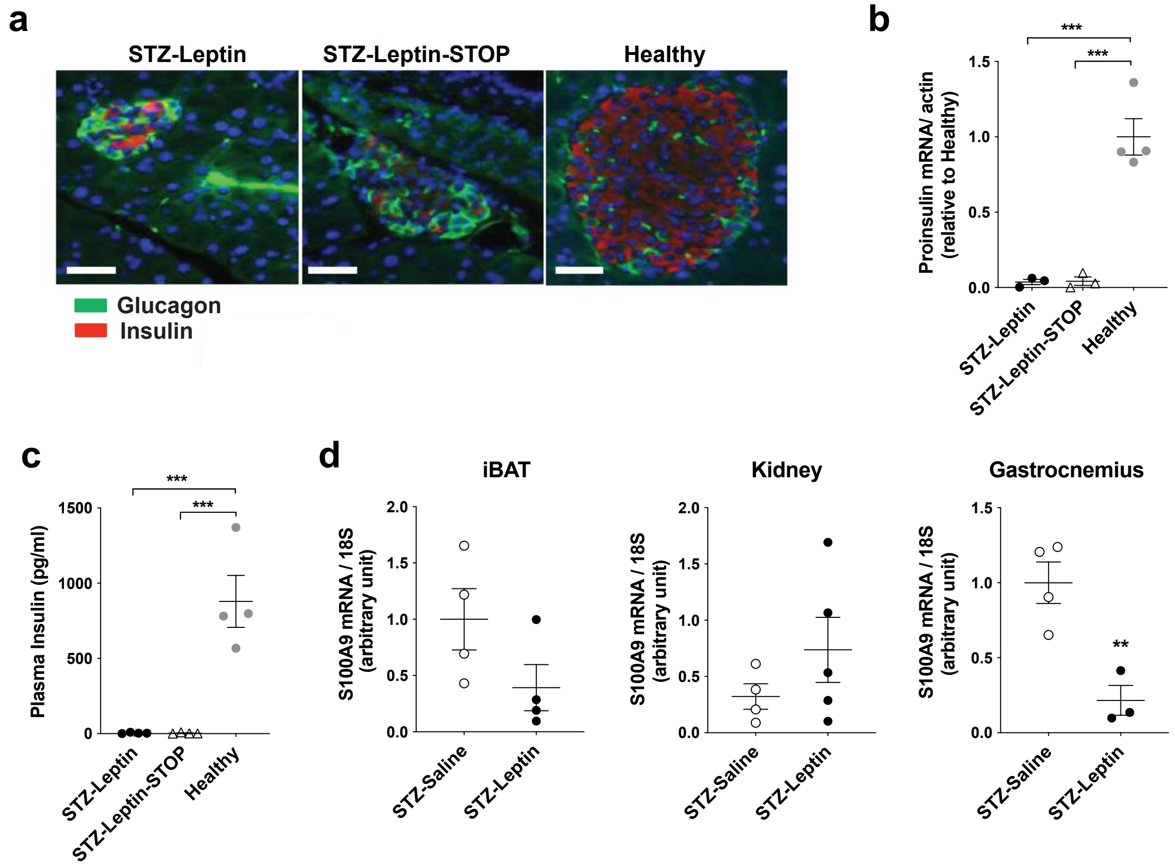

Supplementary Figure 1. Validation of insulin-deficient animal models. Intracerebroventricular (icv) delivery of leptin was performed in two groups of STZ-treated, insulin-deficient mice: STZ-leptin mice received leptin for 12 days; STZ-Leptin-STOP mice received the hormone for 10 days and were followed up to day 12. icv surgery was performed at day 0 and STZ injections were performed 14 and 7 days before surgery. (**a**) Immunohistochemistry of pancreatic islets showing insulin (red) and glucagon (green) in the two aforementioned groups (sacrificed 12 days after icv surgery) and their age-matched non-diabetic healthy controls. Scale bar is 50 µm. In STZ-leptin, STZ-leptin-STOP mice, and their healthy controls: **(b)** pancreatic *Proinsulin* mRNA level (n / group = 3, 3, and 4) and **(c)** plasma insulin levels at day 0 (n / group = 4). **(d)** *S100a9* mRNA level in iBAT, kidney, and gastrocnemius skeletal muscle in a cohort of STZ-treated, insulin-deficient mice that underwent either icv leptin or icv saline treatment for 12 days. mRNA levels of S100A9 were normalized to 18S mRNA levels. Error bars represent SEM (n = 3-4 per group). Statistical analyses were done using a two-tailed unpaired Student's t test or one-way ANOVA (Tukey's post-hoc test) when two or three groups were compared, respectively. **P < 0.01; ***P < 0.001.

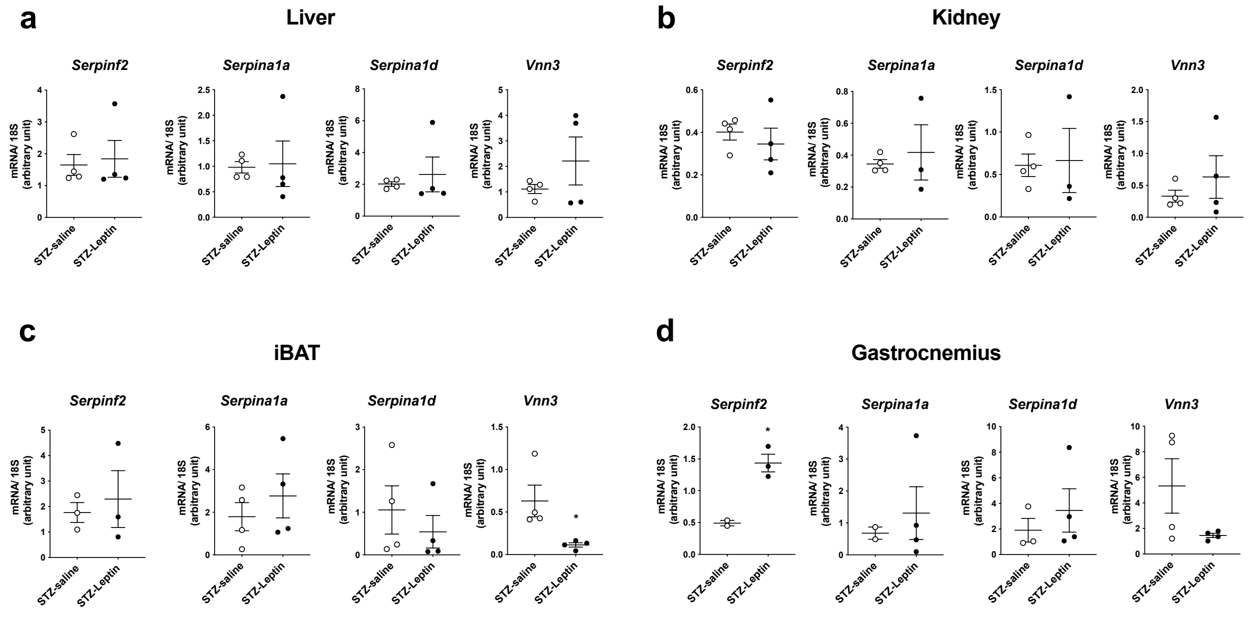

Supplementary Figure 2. Expression of putative leptin-induced cues. *Serpinf2, Serpina1a*, *Serpina1d*, and *Vnn3* mRNA levels were measured in liver (**a**), kidney (**b**), iBAT (**c**), and gastrocnemius skeletal muscle (**d**) in a cohort of STZ-treated, insulin-deficient mice that underwent either icv leptin or icv saline treatment for 12 days. mRNA levels of S100A9 were normalized to 18S mRNA levels. Error bars represent SEM (n / group = 2-4). Statistical analyses were done using a two-tailed unpaired Student's *t* test (Tukey's post-hoc test). *P < 0.05.

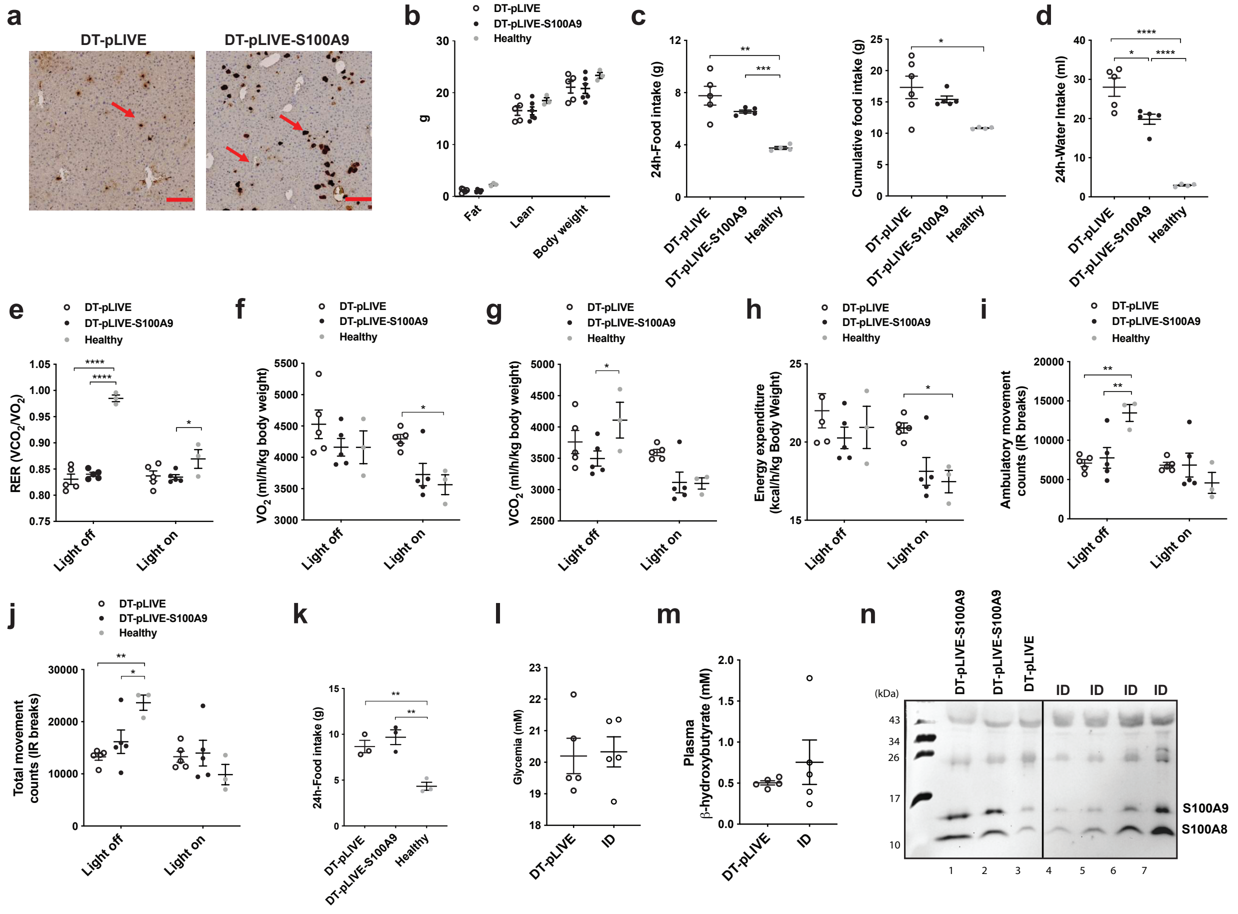

Supplementary Figure 3. Indirect calorimetry in insulin-deficient mice overexpressing S100A9. **(a)** S100A9 immunohistochemistry in liver of DT-pLIVE and DT-pLIVE-S100A9 mice. Arrows indicate endogenous and overexpressing S100A9-positive cells. Scale bar = 100 µm. In DT-pLIVE, DT-pLIVE-S100A9, and their age-matched healthy controls**:** **(b)** body composition (fat and lean mass) and body weight (n /group = 6, 6, and 4); (**c**) daily and cumulative (2.5 days) food and (**d**) water intake (n /group = 5, 5, and 4); (**e**) respiratory exchange ratio (RER), (**f**) oxygen consumption (VO_2), (**g**) carbon dioxide production (VCO_2), (**h**) energy expenditure, (**i**) ambulatory and (**j**) total movements (n /group = 5, 5, and 3). Data were gathered from DT-pLIVE and DT-pLIVE-S100A9 9 days after HTVI injection. (**k**) Daily food intake in cohorts of DT-pLIVE, DT-pLIVE-S100A9, and their age-matched healthy controls. These mice are different from mice shown in (**c**) (n/ group = 3). (**l**) glycemia and (**m**) plasma β-hydroxybutyrate levels of DT-pLIVE mice 9 days after HTVI injection and their DT-treated *RIP-DTR* ID controls (n /group = 5). Error bars represent SEM. Statistical analyses were done using a two-tailed unpaired Student's t test when two groups were compared, and one-way ANOVA or Two-way ANOVA (Tukey's post-hoc test) when more than two groups and more than one experimental condition/time point were compared. $^*P < 0.05$; $^{**}P < 0.01$; $^{***}P < 0.001$, $^{****}P < 0.0001$. (**n**) S100A9/S100A8 ratio was calculated by western analysis of plasma samples from an insulin deficient mouse spiked with increasing concentrations of purified recombinant calprotectin. All samples analyzed in the same gel and S100A8 or S100A9 specific antibodies quantified both subunits. Lane 1: 1µl serum, concentration (c)=10259ng S100A8/A9/ml; ratio S100A9/S100A8 = 0.90; lane 2: 2µl serum, c=5349ng S100A8/A9/ml; ratio S100A9/S100A8 = 1.06; lane 3: 2µl

serum, c=96ng S100A8/A9/ml; ratio S100A9/S100A8 = 0.72, lane 4: 2µl serum, c=78ng S100A8/A9/ml; ratio S100A9/S100A8 = 0.64; lane 5: 2µl serum, c=78ng S100A8/A9/ml + 1 ng calprotectin; ratio S100A9/S100A8 = 0.62; lane 6: 2µl serum, c=78ng S100A8/A9/ml + 5 ng calprotectin; ratio S100A9/S100A8 = 0.48; lane 7: 2µl serum, c=78ng S100A8/A9/ml + 10 ng calprotectin; ratio S100A9/S100A8 = 0.60.

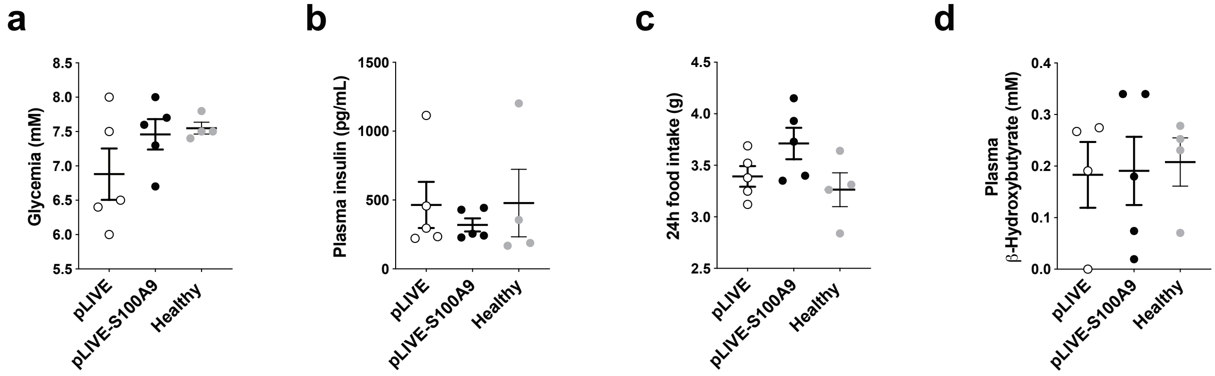

Supplementary Figure 4. Effect of enhanced S100A9 in mice not treated with DT. **(a)** Glycemia **(b)** Plasma insulin content **(c)** 24-hour food intake **(d)** plasma β-hydroxybutyrate in pLIVE and pLIVE-S100A9 (10 days after HTVI) and age-matched healthy controls. Error bars represent SEM. Healthy (n = 4), pLIVE (n = 5) and pLIVE-S100A9 (n = 5). Statistical analyses were done using one-way ANOVA (Tukey's post-hoc test).

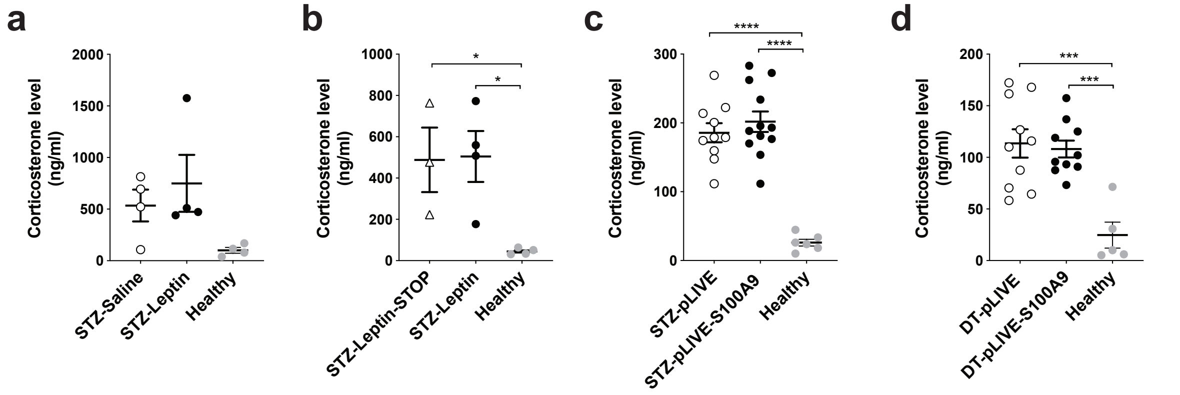

Supplementary Figure 5. Leptin icv treatment or S100A9 overexpression does not improve corticosterone levels in insulin-deficient animals. Plasma corticosterone levels in different insulin-deficient cohorts as compared to their age-matched healthy controls. (**a**) STZ-saline and STZ-leptin mice (12 days after icv surgery), and their healthy controls (n / group = 4, 4, and 4). (**b**) STZ-Leptin-STOP and STZ-leptin mice (12 days after icv surgery), and their healthy controls (n / group = 3, 4, and 4). (**c**) STZ-pLIVE and STZ-S100A9 mice (10 days after HTVI), and their healthy controls (n / group = 10, 12, and 6). (**d**) DT-pLIVE and DT-S100A9 mice (10 days after HTVI), and their healthy controls (n / group = 10, 10, and 5). Error bars represent SEM. Statistical analyses were done using one-way ANOVA (Tukey's post-hoc test). *P < 0.05; ***P < 0.001, ****P < 0.0001.

Gene name	Forward	Reverse
S100a9	ACT CTT TAG CCT TGA AGA GCA AG	TC AGG GTG TCC TTC CTT CC
Serpinf2	CAC AGT GTC GGT GGA CAT GA	CTC GGA CAC GTT CCA CTC AA
Serpina1a	ATA TCC CCC TTG GCT CCC AT	GAG GGA GTC ATT TTC AGC TTG C
Serpina1d	TAA ACA GGC GCA GAA GCG AT	GAG GTC AGC CCC ATT GTT GA
Vnn3	ACA CCC CGT TTG GGA AGT TT	CCC ACA CCG AAT GGA ATG GA
pLIVE For sequencing	GAC TT CTT AAG AGA TGT A	TGG TCT ATA TGG CTC TTG GA
Ins1	GAA GCG TGG CAT TGT GGA T	TGG GCC TTA GTT GCA GTA GTT CT
β-Actin	CAT CGT GGG CCG CCT A	CAC CCA CAT AGG AGT CCT TCT G
18s	ACC GCA GCT AGG AAT AAT GGA	GCC TCA GTT CCG AAA ACC A

Supplementary Table 1. List of primers and sequences.
